# Bioimpedance-defined overhydration predicts survival in end stage kidney failure (ESKF): systematic review and subgroup meta-analysis

**DOI:** 10.1038/s41598-018-21226-y

**Published:** 2018-03-13

**Authors:** Matthew Tabinor, Emma Elphick, Michael Dudson, Chun Shing Kwok, Mark Lambie, Simon J. Davies

**Affiliations:** Institute for Applied Clinical Sciences, Keele University, Newcastle-under-Lyme, UK

## Abstract

Both overhydration and comorbidity predict mortality in end-stage kidney failure (ESKF) but it is not clear whether these are independent of one another. We undertook a systematic review of studies reporting outcomes in adult dialysis patients in which comorbidity and overhydration, quantified by whole body bioimpedance (BI), were reported. PubMed, EMBASE, PsychInfo and the Cochrane trial database were searched (1990–2017). Independent reviewers appraised studies including methodological quality (assessed using QUIPS). Primary outcome was mortality, with secondary outcomes including hospitalisation and cardiovascular events. Of 4028 citations identified, 46 matched inclusion criteria (42 cohorts; 60790 patients; 8187 deaths; 95% haemodialysis/5% peritoneal dialysis). BI measures included phase angle/BI vector (41%), overhydration index (39%) and extra:intracellular water ratio (20%). 38 of 42 cohorts had multivariable survival analyses (MVSA) adjusting for age (92%), gender (66%), diabetes (63%), albumin (58%), inflammation (CRP/IL6–37%), non-BI nutritional markers (24%) and echocardiographic data (8%). BI-defined overhydration (BI-OH) independently predicted mortality in 32 observational cohorts. Meta-analysis revealed overhydration >15% (HR 2.28, 95% CI 1.56–3.34, P < 0.001) and a 1-degree decrease in phase angle (HR 1.74, 95% CI 1.37–2.21, P < 0.001) predicted mortality. BI-OH predicts mortality in dialysis patients independent of the influence of comorbidity.

## Introduction

Observational studies have demonstrated an association between overhydration (OH) and mortality within dialysis patients^1,2^. Fluid status is difficult to assess clinically, risking either hypovolaemia, intradialytic hypotension and loss of residual renal function, or persistent overhydration, manifesting as large interdialytic weight gains, hypertension, left ventricular hypertrophy, peripheral and pulmonary oedema^3^. As such, clinical methods including examination for oedema, minimising interdialytic weight gain or removing fluid until the point of hypotension, known as “probing the dry weight”, are increasingly recognised as inadequate^3^. Gold standard methods in contrast, such as isotope dilution, are more precise, but have been found to be expensive, laborious and not appropriate for mass application in the clinical setting^4^. These limitations have led to the development of bio impedance as a non-invasive, bedside technique to aid the clinical assessment of fluid status and body composition^5,6^. Although based on the same principle, a number of approaches to bioimpedance analysis (BI) of body composition have been developed, including the estimation of bioimpedance vectors and their phase angle at single (typically 50 MHz) or multiple frequencies (bio impedance spectroscopy^3^). Estimates of resistance, inversely proportional to measures of total body water, and reactance, proportional to intracellular mass, can then be used to model body composition^7^. These different approaches express OH in different ways, such as the phase angle (PA), extracellular fluid (ECF) volumes (normalised either to intracellular fluid, ECF/ICF, the total body water, ECF/TBW or height) or derivation of the overhydration index (OH/ECF), defined as the fluid excess or deficit above or below the normally hydrated tissues, especially muscle^3^. Thus a patient may have a raised OH/ECF, i.e. above 15%, yet have a reduced muscle mass (thus TBW).

Overhydration in end-stage renal failure (ESRF) is both a function of salt and water excess and the consequences of reduced muscle mass and abnormal body composition associated with comorbidity and inflammation. Such changes lead to both an absolute and relative expansion of the extracellular fluid volume (ECFv), causing progressive ECFv retention and clinical manifestations of overhydration^3^. The mechanisms underlying cachexia in dialysis patients include chronic inflammation, acidosis, anorexia, insulin resistance, anaemia and metabolic bone disease^8^. Substantial multimorbidity within ESRF patients precipitates physical weakness and deconditioning, further exacerbating muscle wasting^8^. Given that observational studies commonly measure hypoalbuminaemia, inflammation and the presence of comorbidities, which themselves predict mortality within the dialysis population, it is important to establish whether overhydration, as determined from body composition measurements, is an independent predictor of survival.

The purpose of this review is to summarise the evidence regarding the use of whole-body BI in dialysis patients to explore whether BI-defined overhydration (BI-OH) is independently predictive of mortality. Where possible, quantitative pooling of outcome data was planned to determine the extent to which BI-OH can be treated as a mortality predictor. Secondary analyses were planned to explore whether BI-OH is predictive of morbidity related markers, such as re-hospitalisation. Studies invesitgating the role of whole-body BI-OH in heart failure (HF) were included to compare whether the relationship is consistent across different chronic disease groups.

## Methods

### Study design

The design and reporting of the systematic review protocol was guided by the Preferred Reporting Items for Systematic Reviews and Meta-analysis (PRISMA)^9^. All studies reporting primary (mortality) or secondary (re-hospitalisation/morbidity measures) outcome data, in adults with ESRF or HF, where whole-body BI measurements or degree of overhydration was specified, were considered appropriate for inclusion within the study.

### Search Strategy and Selection Criteria

MEDLINE, EMBASE, PsychINFO and the Cochrane Register for Controlled Trials (CENTRAL) were searched from 01/01/1990 through to 06/11/2017 to identify relevant citations. 1990 was chosen as the lower cut off as BI machines were not routinely available prior to this date. Search terms included both medical subject headings (MeSH) and agreed *a priori* individual search terms. Reference lists from identified citations and selected manual journal searching was used to identify any further relevant studies that matched the inclusion criteria prior to data extraction. The search strategy used for CENTRAL is available as an appendix (see supplementary material).

### Study Selection and Data Extraction

All retrieved citations were imported from the citation library into a central database (using Microsoft Excel 2011). Citations were assessed at the title and abstract level by two independent reviewers (MT and EE or SJD) using exclusion criteria: the study was in the paediatric population; the wrong BI measure was used (i.e. segmental and intrathoracic BI methods); the outcome of interest for our review was not reported (i.e. mortality and hospitalisation); there was no full paper available; or there was no English translation available. During abstract review, if the citation suggested that the study assessed prognostic outcome data, or if it was unclear from the abstract what the study outcomes were, then the citation was accepted for full paper review. Full paper review was conducted again using two independent reviewers (MT and MD or SJD), using the same exclusion criteria. Data extraction occurred both at the individual study level, using piloted study summaries (on Microsoft Word 2011), and in the form of review summary tables (on Excel 2011). No *a priori* assumptions were made regarding data quality.

### Classification of BI method for expressing fluid status

Studies were sub-grouped according to whether they used phase angle (PA)/BI-vector analysis (BIVA), normalised ECF (ECF/TBW) or the overhydration index (OHI) as previously described^3^.

### Risk of Bias (ROB) Assessment

Risk of bias (ROB) within studies was assessed by two independent reviewers (MT and MD or SJD) using the Quality in Prognostic Studies (QUIPS) tool^10^, which grades six separate study domains (selection of participants, study attrition, prognostic factor measurement, outcome measurement, study confounding and statistical analyses) according to their risk of Bias (low, medium or high risk of bias). If disagreements occurred then this was resolved following discussion between MT and MD.

### Statistical Analyses and Meta Analysis Methods

All cohorts reporting multivariable survival analyses (MVSA) for outcome data (mortality odds ratio, risk ratio or hazard ratio with 95% confidence intervals) were considered for quantitative pooling in a meta-analysis. More than one cohort had to report the same BI-OH method and cut off value defining overhydration within MVSA to be included for pooling. In all PA cohorts mortality hazard ratios were expressed for every 1-degree increase in PA in initial MVSA. Therefore, to better reflect the effect of increasing overhydration defined by PA on mortality, individual cohort mortality HR and 95% confidence intervals were mathematically reciprocated before pooled summaries were produced. Random effects pooled summaries, using the generic inverse variance method, were produced using Review Manager 5.3 (Nordic Cochrane Centre). The I^2^ statistic was used to assess statistical heterogeneity, with I^2^ values between 30%–60% representing moderate levels of heterogeneity^11^.

### Data availability

The datasets generated during the current study are available from the corresponding author on reasonable request, including the full search strategy used to identify citations and the QUIPS paper summaries.

## Results

**Figure 1:**
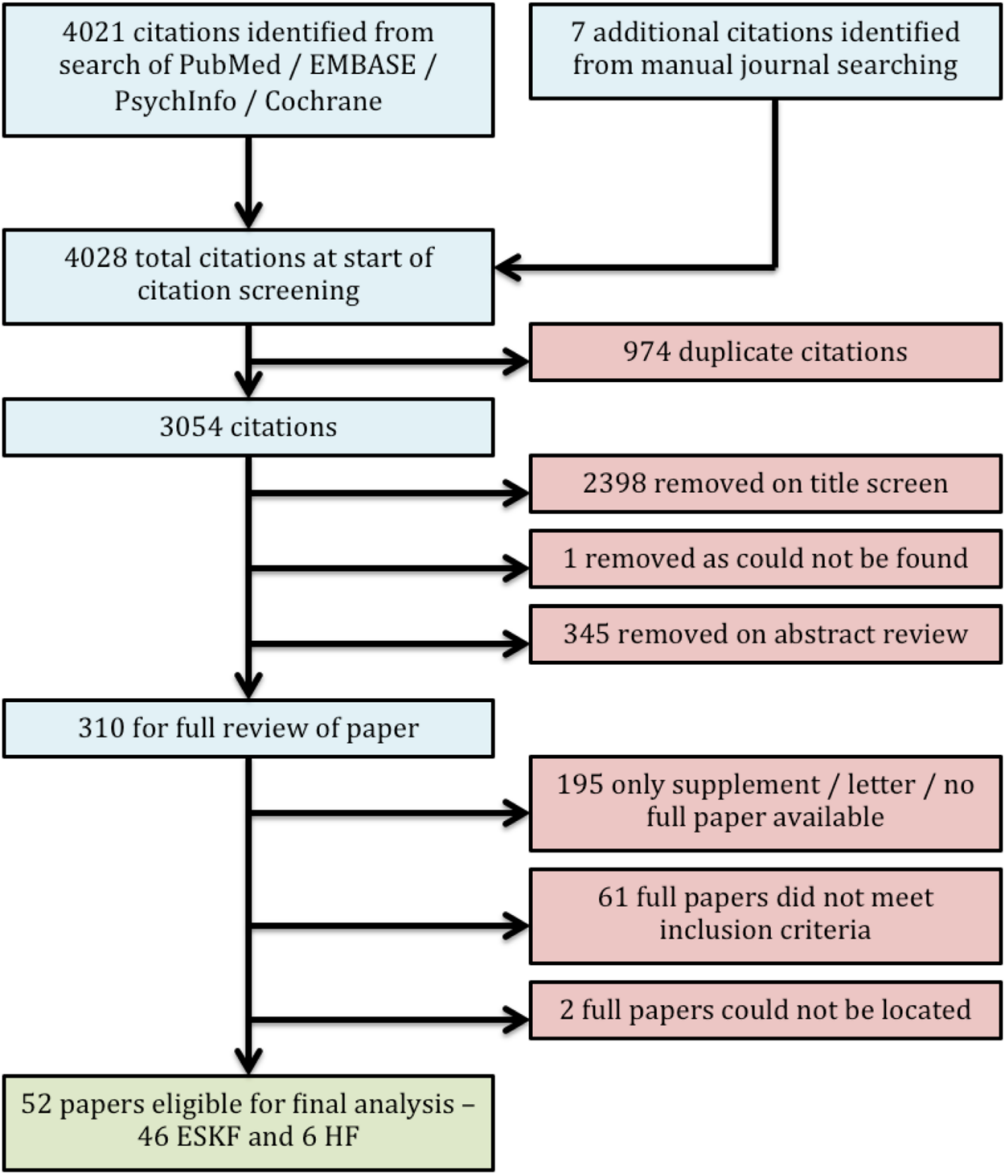
PRISMA flow diagram. The flow diagram summarises the systematic search, citation screening, exclusion and inclusion processes undertaken within this review.

The search yielded 3054 citations following removal of duplicates and identification of 7 additional citations from selected manual journal searching and reference checking (Fig. 1). Detailed methodological review and data extraction process was conducted on 52 papers;^1,2,12–61^ 46 from the ESKF population^1,2,12,14–17,19,20,22–25,27–50,52–59,61^and 6 from the HF population^13,18,21,26,51,60^. Within ESKF studies 42 individual cohorts were identified, with 4 cohorts having data reported in two separate studies.

### Heterogeneity within the use of BI measurements of hydration status

**Figure 2:**
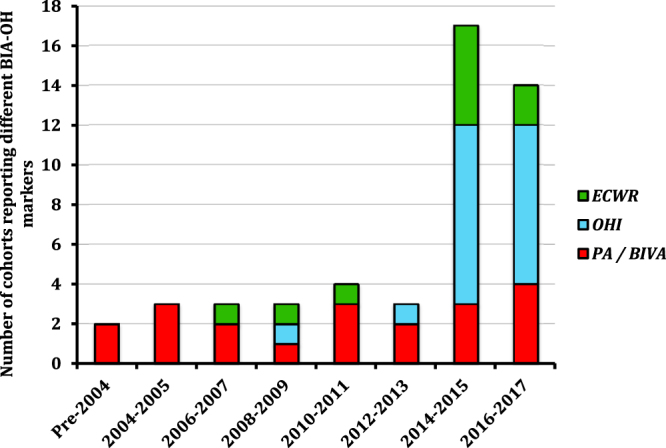
The temporal change in the reporting of BI-OH measures within studies according to year of publication. PA = phase angle, BIVA = Bioimpedance Vector Analysis, ECWR = Extracellular water expressed as a ratio (e.g. intracellular or total body water, OHI = overhydration index.

The most common BI-OH measure, phase angle (PA) and the related measure of BI-vector analysis (BIVA), was reported in 41% of studies^12–16,18,20–22,24–26,29,30,38,39,47,49,52–54,60^. Overhydration indices (OHI), as a group, were reported in 39% of studies^1,2,17,23,33–35,37,40–44,46,48,55–58,61^. OHI was variably described, with relative fluid overload (RFO), overhydration normalised for extracellular water (OH/ECW), absolute overhydration in litres and the percentage hyperhydration compared to normally hydrated controls all being reported. Extracellular water ratios (ECWR) were reported in 20% of studies^19,22,27,28,31,32,36,41,45,50,51,59^, with ECW ratios being variably normalised for intracellular water (ECW/ICW), total body water (ECW/TBW) and body surface area (ECW/BSA). The changes in the use of different BI-OH measures over time are summarised in Fig. 2.

### Patient demographics within ESKF and HF cohorts

60,790 ESKF patients were identified in 42 separate cohorts (Table 1); cohort size ranged between 45 to 39,566 patients and within all cohorts 8,168 mortalities were reported. 57,563 patients were on haemodialysis (95%) and 3227 on peritoneal dialysis (5%). 20 (48%) cohorts were within Europe^1,2,12,17,25,27,30,33,35,39,41,42,46,48,52,53,55–58^, 5 (12%) within the USA, 9 (21%) within South East Asia, 2 (5%) within Central/South America, 3 (7%) within the Middle East^15,16,24^ and 1 (2%) within the Indian Subcontinent^40^. 2 cohorts (5%) were multinational studies^23,61^. Follow up ranged from 0–15 years (1 study stating a follow up of 339 years, which was thought to represent cumulative follow up^25^). Patient age, gender, ethnicity and diabetic status were reported in 100, 98, 29 and 90% of cohorts respectively. Mean age ranged between 38 to 69 years^1,2,12,14–17,19,20,22–25,27–50,52–59,61^, the proportion of males between 31–79%^1,12,14–17,19,20,22–25,27–50,52–59,61^, the proportion of non-Caucasian patients between 2 to 100%^14,20,28–30,38,41,42,46,47,49,58,61^ and the proportion of patients who were diabetic between 8 to 58%^1,12,14–17,19,20,22–25,27,29–36,38,40–48,50,52–59,61^. HIV status was reported in 1 cohort^28^, and was cited as exclusion criteria in 6 others^20,22,29,38,47,49^. Primary renal disease (PRD) was described in 48% of cohorts^12,16,22,25,29,30,37–39,41,43,44,46,49,50,56^, with diabetic nephropathy (55%) and glomerulonephritis (30%) being the commonest aetiology respectively.

**Table 1:** Summary of studies fulfilling the search criteria.

Author(s)	Year	Country	RRT	N	Follow up	Prim. Outcome	Sec. Outcome	N (Mort)	QUIPS Criteria						BIA Method	Outcome of Study (with appropriate MVA outputs, if present)
									SP	SA	PFM	OM	SC	SAR		
**End-Stage Kidney Failure Cohorts**																
Abad	2011	Spain	HD+PD	164	6 yrs	Mortality	N/A	100	L	M	L	L	L	M	PA*	PA < 8 (p < 0.01) and comorbidity (Charlson index) are independent predictors of mortality
Avram	2006	USA	PD	177	15 yrs	Mortality	N/A	89	H	H	M	M	H	H	PA*	PA (RR 0.54) and enrolment CRP were independent predictors of mortality.
Bebera-shvili	2014	Israel	HD	91	3 yrs	Mortality	N/A	38	L	L	L	L	M	L	PA**	Patients with greatest decline of PA had highest risk for mortality. 1 degree increase in PA (when treated as time varying variable) has a mortality HR of 0.61 (95% CI 0.53–0.71)
Bebera-shvili	2014	Israel	HD	250	1.4 yrs	Mortality	N/A	64	L	L	L	L	M	M	PA***	As a continuous variable PA has a mortality HR of 0.72 (95% CI 0.54–0.96). Adjustment for MIS Score nullified of PA as mortality predictor (HR 0.75, 95% CI 0.54–1.03) but remained a predictor of hospitalisation risk (HR 0,76, 95% CI 0.63–0.92)
Caetano	2016	Portugal	HD	697	1 yr	Mortality	N/A	66	M	L	L	M	L	M	OHI**	OH/ECW >15% is an independent predictor of 1-year mortality during follow up (HR for mortality 2.22, 95% CI 1.29–3.79).
Chazot	2012	France	HD	158	6.5 yrs	Mortality	Hypertension	Unclear	M	L	L	L	H	M	OHI**	Giessen cohort patients with hyperdration (OH/ECW >15%) had worse cumulative survival than non-hyperhydrated patients (mortality HR 3.41, 95% CI 1.62–7.17).
Chen	2007	China	PD	227	3 yrs	Mortality	N/A	58	L	L	M	L	L	M	ECWR**	ECW/ICW is independent predicator of mortality in incident PD patients. Every 0.1 increase in ECW/ICW (time dependent) associated with mortality RR 1.37 (95% CI 1.10–1.70)
de Araujo	2013	Brazil	HD+PD	145	1.3 yrs	CV Events	Mortality	13	M	L	M	L	L	L	PA	PA is predictive of CV events in non-diabetics (HR 0.56, 95% CI 0.38–0.83) but not in diabetics (HR 1.01, 95% CI 0.60–1.70). Study has small number of endpoints.
Dekker	2017	Inter-national	HD	8883	1 yr	Mortality	N/A	Unclear	L	L	M	M	M	H	OHI**	Baseline pre-dialysis OHI/ECW >15% (overhydration 2.5–5L) is predictive of mortality (HR 2.62, 2.1–3.3), independent of comorbidity. Inflammation and FO in a dynamic cohort have additive effects on mortality in HD patients.
Demirci	2016	Turkey	HD	493	2.3 yrs	Mortality	CV Mortality	93	L	L	L	L	M	M	BIVA**	When adjusted for comorbidities, impedance ratio is independently predictive for all cause mortality (HR 1.13, 95% CI 1.04–1.23) and cardiovascular mortality (HR 1.15, 95% CI 1.03–1.27)
Di Iorio	2004	Italy	HD	515	339 yrs^	Mortality	N/A	75	L	M	M	M	L	M	PA*	PA was an independent predictor of mortality in a HD population (RR 2.50).
Fan	2015	UK	PD	183	1.7 yrs	Mortality	Technique Failure	37	L	M	M	L	M	L	ECWR**	In PD patients, ECW is an independent predictor of mortality, including in cases adjusted for peritonitis episodes (HR 2.98, 95% CI 1.40–7.30). Log CRP also an independent predictor of mortality (HR 3.32, 95% CI 1.50–7.70).
Fein	2002	USA	PD	45	0.6 yrs	Mortality	N/A	4	L	M	M	M	H	H	PA****	Univariate analysis revealed patients with PA < 6 had worse cumulative survival than those >6 (p < 0.01). No MVA present.
Fein	2008	USA	PD	53	8 yrs	Mortality	N/A	21	H	H	L	M	M	M	ECWR*	Enrolement BIA measures (Avram *et al*., ECM/BCM, RR 1.04/Fein *et al*., ECW/BSA, RR 1.50) were independent predictors of mortality. Avram *et al*. data included as same cohort*
Fiedler	2009	Germany	HD	90	3 yrs	Mortality	Hospital admission events	36	M	L	M	L	M	L	PA**	PA < 4 independently predicts mortality in HD patients (RR 2.34, 95% CI 1.06–5.14). Individual nutrition scores are superior to BIA in terms of prognostic utility.
Guo/Guo	2015	China	PD	307	3.2 yrs	Mortality	CV Mortality	52	L	L	L	L	M	L	ECWR**	In CAPD patients ECW/TBW >0.40 is an independent predictor of all cause mortality (HR 13.12, 95% CI 1.35–128.00) and PD technique failure (HR 10.34, 95% CI 1.88–57.02).
Hoppe	2015	Poland	HD	241	2.5 yrs	Mortality	N/A	42	M	H	H	M	H	H	OHI	Troponin and OH index predict mortality in 1 MVA, but when adjusted for other covariates, OH Index (continous variable) no longer an independent predictor of mortality (RR 1.12, 95% CI 0.92–1.37).
Huan-Sheng	2016	Taiwan	HD	298	1 yr	Hospital admission events	CV Events	13	**QUIPS not validated for assessing bias in RCT**						OHI	No differences noted in all cause hospitalisation (HR 1.19, 95% CI 0.79–1.80), all cause mortality (HR 0.85, 95% CI 0.29–2.53) and fluid overload/cardiovascular event rate (HR 0,57, 95% CI 0.08–1.07) between the BIA and control groups.
Jotterand-Drepper	2016	Germany	PD	54	6.5 yrs	Mortality	N/A	19	L	L	M	L	M	H	OHI**	OHI/ECW >15% independently predictive of mortality (HR 7.82, 95% CI 1.10–29.07) in PD patients when adjusted for troponin values, CRP, the presence of heart failure and hypoalbuminaemia.
Kim	2015	South Korea	HD	240	2 yrs	Mortality	Hospital admission events	50	M	M	L	L	M	M	OHI**	When adjusted for comorbidities, OH/ECW >15% was an independent predictor of mortality (HR 2.58, 95% CI 1.16–5.75). Age was also an independent predictor.
Kim	2017	South Korea	HD	77	5 yrs	Mortality	CV Events	24	L	L	H	L	M	M	ECWR**	ECW/ICW ratio is an independent predictor of mortality (HR 1.12, 95% CI 1.01–1.25) and cardiovascular events (HR 1.09, 95% CI 1.01–1.18) when adjusted for multiple co-morbidities.
Koh	2011	Malaysia	PD	128	2.2–2.3 yrs	Mortality	N/A	35	L	M	L	L	L	M	PA**	PA is a independent predictor of mortality in HD patients (HR 0.39, 95% CI 0.27–0.57).
Maggiore	1996	Italy	HD	131	2.2 yrs	Mortality	N/A	23	M	L	L	L	H	M	PA*	When adjusting for age and other nutritional markers, PA is an independent predictor of mortality in HD patients (p < 0.01).
Mathew	2015	India	HD+PD	99	2 yrs	Mortality	N/A	33	M	L	L	L	M	L	OHI**	Absolute overhydration (>3.1L) is an independent predictor of mortality (adjusted OR 2.96, 95% CI 1.04–8.46).
O’Lone	2014	UK	PD	529	4 yrs	Mortality	N/A	95	M	M	L	L	H	L	OHI+ECWR**	Where OH/ECW and ECW/TBW values are in the top 30% for the cohort, both OH/ECW (HR 2.09, 95% CI 1.36–3.20) and ECW/TBW (HR 2.05, 95% CI 1.31–3.22) act as independent predictors of mortality.
Oei	2016	UK	PD	336	2 yrs	Mortality	N/A	48	L	L	M	L	H	M	OHI****	Univariate analysis correlates overhydration with cardiac death (p < 0.05), but no further analysis noted.
Onofriescu	2014	Romania	HD	131	3.5 yrs	Mortality	Adverse Events	9	**QUIPS not validated for assessing bias in RCT**						OHI**	RCT of BIA vs standard clinical care in determining ultrafiltration on HD. BIA group had survival advantage over standard clinical care group (HR 0.11, 95% CI 0.01–0.92). Study at risk of selection bias.
Onofriescu	2015	Romania	HD	221	5.5 yrs	Mortality	CV Mortality	66	L	L	M	L	L	L	OHI**	OH/ECW >17.4%, when adjusted for comorbidities, is independently predictive for mortality when LVEF (HR 2.29, 95% CI 1.08–4.89) and LVMI (HR 2.19, 95% CI 1.02–4.69) are adjusted for in the analysis.
Paniagua	2010	Mexico	HD+PD	753	1.4 yrs	Mortality	CV Mortality	182	M	L	M	L	M	L	ECWR**	ECW/TBW (OR 1171.33, 95% CI 3.35–409899.37) and NT-proBNP (OR 1.01, 95% CI 1.00–1.02) independently predictive of CV mortality but not all cause mortality (OR 84.64, 95% CI 0.52–13788.55) in dialysis patients.
Paudel	2015	UK	PD	455	2 yrs	Mortality	N/A	72	L	H	M	M	L	H	OHI****	Univariate analysis revealed OH index predictive of mortality. Multivariable model used to assess SGA independent of OH.
Pillon/Chertow	2004	USA	HD	3009	0–1.5 yrs	Mortality	N/A	361	M	L	L	M	M	M	BIVA**	BIVA vector, per 100Ohm/m incremental increase, is independently predictive of mortality (RR 0.75, 95% 0.57–0.88).
Ponce	2014	Portugal	HD	189	1 yr	Mortality	Adverse Events	20	**QUIPS not validated for assessing bias in RCT**						OHI	Univariate analysis revealed survival (p = 0.33) and event-free-survival (p = 0.17) equivalent between BIA and control groups. The study was terminated prematurely.
Pupim	2004	USA	HD	194	3 yrs	Mortality	CV Mortality	50	M	M	L	L	M	L	PA*	PA and Albumin independent predictors of cardiovascular mortality in MVA, although summary statistics from MVA not reported.
Rhee	2015	South Korea	PD	129	2.1 yrs	Residual RF	Mortality	15	M	H	L	M	M	H	ECWR**	In Korean PD patients with preserved RRF, ECW/TBW is predictive of mortality (HR 1.001, 95% CI 1.001–1.086) and, additionally, technique failure (HR 1.024, 95% CI 1.001–1.048).
Segall/Segall	2014	Romania	HD	149	1.1 yrs	Mortality	N/A	43	L	M	M	M	L	L	PA**	PA < 5.58 is independently predictor of mortality in HD patients (HR 2.15, 95% CI 1.16–3.99).
Shin	2017	South Korea	HD	142	2.4 yrs	Mortality	CV Mortality	15	L	M	M	M	H	M	PA**	PA is an independent predictor of all cause mortality (HR 0.56, 95% CI 0.33–0.97) and infection (HR 0.65, 95% CI 0.45–0.94) in HD patients, but not for cardiovascular mortality (HR 0.92, 95% CI 0.43–2.14).
Siriopol/Siriopol	2015	Romania	HD	173	1.8 yrs	Mortality	N/A	31	L	M	M	L	H	H	OHI**	OH/ECW >6.68% (HR2.93, 95% CI 1.30–6.58) and lung comet score (LCS>22; HR 2.72, 95% CI 1.19–6.16) independently predictive of mortality. Earlier study (2013) OH/ECW not predictive of mortality but was underpowered.
Siriopol	2017	Romania	HD	285	3.4 yr	Mortality	N/A	89	L	L	M	L	M	L	OHI	In combination overhydration (>6.9%) and high NT-proBNP levels independently predict mortality (HR 1.83, CI 1.02–3.54, whereas in patients with normal NT-proBNP levels overhydration is not a predictor (HR 1.34, 95% CI 0.67–2.68)
Tangvora-phonkchai	2016	UK	HD	362	4.1 yr	Mortality	N/A	110	L	L	L	M	L	L	OHI**	OH (%, as a continuous variable) is an independent predictor of mortality in MVSA (HR 1.15, 95% CI 1.03–1.28); co-morbidity, non-BIA nutritional indices, albumin and CRP also noted to be independent predictors of mortality.
Tian	2016	China	PD	152	5 yrs	Mortality	N/A	44	L	M	L	M	M	M	ECWR	When adjusted for inflammation (CRP), ECWR (a standard deviation away from the median) is not predictive of mortality in PD patients (HR 2.20, 95% CI 0.79–6.08)
Wizemann	2009	Poland	HD	269	3.5 yrs	Mortality	N/A	86	L	L	L	M	L	M	OHI**	OH/ECW>15% is an independent predictor of mortality in HD patients (HR 2.10, 95% CI 1.39–3.18).
Zoccali	2017	International	HD	39566	1.4 yrs	Mortality	N/A	5866	L	M	L	M	L	L	OHI**	Baseline OHI/ECW>15% (men)/13% (women) at baseline independent predictor of mortality (HR 1.26, 95% CI 1.19–1.33) and in patients with cumulative fluid overload over a 1 yr period irrespective of pre-dialysis systolic BP.
**Heart Failure Cohorts**																
Alves	2016	Brazil	N/A	71	2 yrs	Mortality	N/A	34	L	M	L	L	H	M	PA**	PA < 4.8 independent predictor of mortality in following episodes of acute decompensated heart failure (HR 2.67, 95% CI 1.21–5.89). Ejection fraction also independent predictor of mortality (HR 0.94, 95% CI 0.89–1.00)
Castillo-Martinez	2007	Mexico	N/A	242	N/A	NYHA Class	N/A	Unclear	L	H	M	H	H	M	PA + BIVA****	Univariate analysis demonstrated PA predicts severity of symptoms (indirect measure of risk of hospitalisation) in both HFSD + HFPSF.
Colin-Ramirez	2012	Mexico	N/A	389	3 yrs	Mortality	NYHA Class	66	L	L	L	L	M	M	PA**	Following adjustment for age, haemoglobin and diabetic status, a PA < 4.2 was independently predictive of all cause mortality (HR 3.08, 95% CI 1.06–8.99).
Doesch	2010	Germany	N/A	41	5 yrs	Cardiac MRI data	CV Mortality	8	M	M	L	M	M	L	PA	On univariate analysis, PA>5.5 correlated with CV survival, but not statistically significant (p = 0.13).
Sakaguchi	2015	Japan	N/A	130	0.5 yrs	Adverse Events	CV Events	37 (2 deaths)	L	L	L	L	M	L	ECWR**	In acute decompensated heart failure, ECW ratio (measured/predicted) independent predictor of cardiac death/re-admission (HR 1.48, 95% CI 1.20–1.83).
Trejo-Velasco	2016	Spain	N/A	105	0.9 yrs	Mortality	Readmission	19	M	M	L	L	H	M	BIVA**	Hyperhydration (defined by BIVA readings>74.3%) was an independent predictor of adverse outcomes (HR 2.6, 95% CI 1.1–6.4),

Individual patient cohorts listed according to author(s), year of publication (where multiple studies from the same cohort are identified, lead authors of each study and year of most recent study cited) and geographical location of cohort. For each cohort, the BI-OH markers, dialysis modalities, follow up period, number of patients within the cohort, primary/secondary outcomes, number of endpoints and summary of findings are provided. Summaries for each cohort are given, along with the appropriate BI-OH marker and its utility within the cohort to predict survival (denoted by the numbers of * by the BI-OH marker): * shows that the BI-OH marker is an independent predictor of the primary outcome (but does not report a hazard ratio/risk ratio/odds ratio and confidence interval), ** shows the BI-OH marker is an independent predictor of the primary outcome (and reports hazard ratio/risk ratio/odds ratio and confidence interval), *** shows the BI-OH is an independent predictor of secondary but not primary outcome and **** shows that BI-OH is a univariate predictor of primary outcome. QUIPS risk of Bias summaries are provided for each cohort, with QUIPS domains coded as “L” for low risk of bias, “M” as medium risk of bias and “H” as high risk of bias. QUIPS domains include SP = Study participation, SA = Study attrition, PFM = Prognostic factor measurement, OM = Outcome measure, SC = Study confounding and SAR = Statistical analysis reporting. Randomised controlled trials (RCT) were not quality appraised using QUIPS as this is not a valid method of appraising methodological quality in this study design. In one study (highlighted ^), the follow up period was reported ambiguously and may have reflected cumulative follow up.

978 HF patients with were identified in 6 separate cohorts (Table 1); cohort size ranged between 41 to 389 patients and within all cohorts 164 primary endpoints (129 mortalities/35 re-hospitalisations for heart failure) were identified. 2 (33%) cohorts were within Europe^26,60^, 1 (17%) within South East Asia^51^ and 3 (50%) within Central/South America^13,18,21^. Follow up ranged from 0–5 years. Patient age, gender, ethnicity and diabetic status were reported in 100, 100, 17 and 66% of cohorts respectively. Mean age ranged from 59 to 74 years^13,18,21,26,51,60^, the proportion of males from 39 to 88%^13,18,21,26,51,60^ and the proportion of patients with diabetes between 37–59%^18,21,51,60^.

### BI-defined overhydration, mortality and morbidity in ESKF

Within 42 ESRF cohorts, BI-OH was associated with an increased risk of mortality in 35 (83%). 39 cohorts were observational cohorts (Table 1). All 3 observational cohorts with univariate analyses demonstrated that BI-OH is predictive of mortality in the PD population^29,42,46^. Within 36 observational cohorts containing a multivariable survival analysis (MVSA), 31 demonstrated that BI-OH is an independent predictor of all cause^1,2,12,14,15,17,19,20,23–25,27,28,30–32,35–41,44,45,47,50,52–56,58,61^ and cardiovascular mortality^49^, with a further cohort demonstrated BI-OH as an independent predictor of hospitalisation^16^. 3 cohorts were randomised controlled trials (RCTs) assessing the role of BI versus standard care in determining fluid status in HD patients^34,43,48^. In the RCTs using a MVSA, 1 demonstrated using BI-OH provided independent survival benefit^43^ whereas 1 did not^34^. Details of covariates included in MVSA within each cohort are listed in supplementary Table 1. Looking at all cohorts, the adjusted covariates included demographics, such as age (92%^1,2,12,14–17,19,20,22–24,27,28,30–32,34–39,41,43–45,47,49,50,52–59,61^), ethnicity (16%^14,20,28,41,47,61^) and gender (66%^1,2,12,14–17,19,20,23,24,28,30–32,34,37,38,41,43–45,47,49,50,54,57^); co-morbidities, such as diabetic status (63%^1,2,14–17,20,23,24,27,28,30–33,36–38,41,43–45,47,50,52,53,61^), hypertension (32%^1,2,22,24,31,32,35,36,38,44,45,59,61^), cardiovascular disease (24%^2,15,23,24,36,37,43,44,61^), BMI (21%^1,17,23,24,40,43,52,53,61^), heart failure (11%^23,35,52,53,61^), HIV status (3%^28^) and co-morbidity scoring systems (21%^12,16,19,25,31,32,41,54,58^); dialysis related factors such as dialysis vintage (50%^1,2,12,15–17,20,23,24,28,30,38,41,43–45,47,52,53,55–57^), dialysis modality (5%^22,45^), Kt/V (29%^2,16,19,20,24,31,32,37,38,47,49,50,61^), peritoneal solute transport rate (3%^31,32^) and residual renal function (5%^16,31,32^); biochemical factors such as CRP (34%^22,27,30–32,35,41,45,49,55–59,61^), IL-6 (3%^16^), cholesterol (5%^20,33^), HbA/haematinics (18%^1,20,24,25,37,47,57,61^), albumin (58%^1,2,17,19,20,24,25,27,30–33,35,37,38,41,45,47,49,50,55,56,58,59,61^), phosphate (13%^2,37,38,57,61^) and BNP (11%^2,45,57,61^); non-BI nutritional markers (24%^16,19,23,30,39,52,53,58,59,61^); echocardiographic markers such as left ventricular ejection fraction (3%^44^) and markers of left/interventricular wall thickness (8%^33,44,55,56^); symptomatic markers, such as NYHA classification of dyspnoea (5%^55–57^); and the duration of hospital admission (3%^25^).

In four observational studies using MVSA BI-OH did not independently predict adverse outcomes^22,33,57,59^. A Brazilian cohort demonstrated BI-OH was predictive of cardiovascular (CV) event rate in diabetics but not in non-diabetics^22^. This study, however, was relatively underpowered and reported cardiovascular event rate as opposed to mortality. A Polish cohort determined the effect of dialysis vintage on survival, with secondary analyses exploring the effects of BI-OH, echocardiographic data and troponin levels on survival^33^. In this analysis, which included the cardiac biomarker troponin, BI-OH did independently predict mortality, but when adjusted for albumin, cholesterol and intraventricular septum thickness on echocardiography, this relationship was not seen. A Romanian cohort, which explored the additive value of BNP and relative overhydration (ROH) in predicting mortality in HD patients^57^ found that while these had an additive effect in predictiting survival, ROH alone was not an independent predictor, possibly due to relatively small numbers in this sub-group analysis. In a Chinese cohort of PD patients, increased extra-intra cellular water ratio was predictice of worse survival in MVSA, except of r the final models which incorporated C-reactive protein; again this was likely underpowered given the number of covariates used and low number of deaths^59^.

### Association of BI-defined overhydration with echocardiography, cardiac biomarkers and survival in ESKD

Three cohorts explored the relationship between BI-OH and cardiac function. Two cohorts demonstrated that BI-OH independently predicted all-cause mortality^44,55,56^ and cardiovascular events^44^ when adjusted for left ventricular ejection fraction (LVEF^44^) and left ventricular mass (LVM^44,56^). In the Onofriescu *et al*. cohort, both LVEF and LVM were measured by a blinded cardiologist. Within the MVSA, relative overhydration >17.4% independently predicted mortality following adjustment for both LVEF and LVM^44^. In the Siropiol *et al*. cohort pre-dialysis BI-OH was used in conjunction with post-dialysis LVM and ultrasound assessment of pulmonary congestion (comet scores). Following adjustment for comorbidities and LVM, BI-OH and ultrasound comet scores were both independently predictive of mortality, with the BI-OH marker (OH/ECW > 6.68%) being the superior predictor^56^. Siropol *et al*. previously demonstrated that LVM was and BI-OH/LVEF were not independently predictive of mortality in dialysis patients, but compared with the their later work this study was underpowered^55^. Four cohorts adjusted for BNP in MVSA; 1 study demonstrated both BNP and BI-OH as a independent predictors of mortality^45^, 2 studies demonstrated that BI-OH was predictive of mortality whereas BNP was not^35,58^ and 1 study demonstrated an additive effect^57^.

### Association of BI-defined overhydration with morbidity and mortality in Heart Failure

Five heart failure cohorts demonstrated an association between BI-OH and adverse patient outcomes (^13,18,21,51,60^, Table 1). One cohort undertook univariate analyses and demonstrated BI-OH values predicted the severity of HF symptoms (NHYA classification) in heart failure^18^. Four cohorts included MVSA, adjusting for covariates such as age (100% of MVSA), diabetic status (25%), renal dysfunction (50%), LVEF (50%), BNP (25%) and haemoglobin/haematinics (50%). In all four cohorts with a MVSA BI-OH was independently predictive of all cause mortality^13,21,60^ and adverse events^51^.

### Quantitative pooling (sub-group meta-analysis) of BI-OH as a predictor of mortality in ESKF cohorts

**Figure 3:**
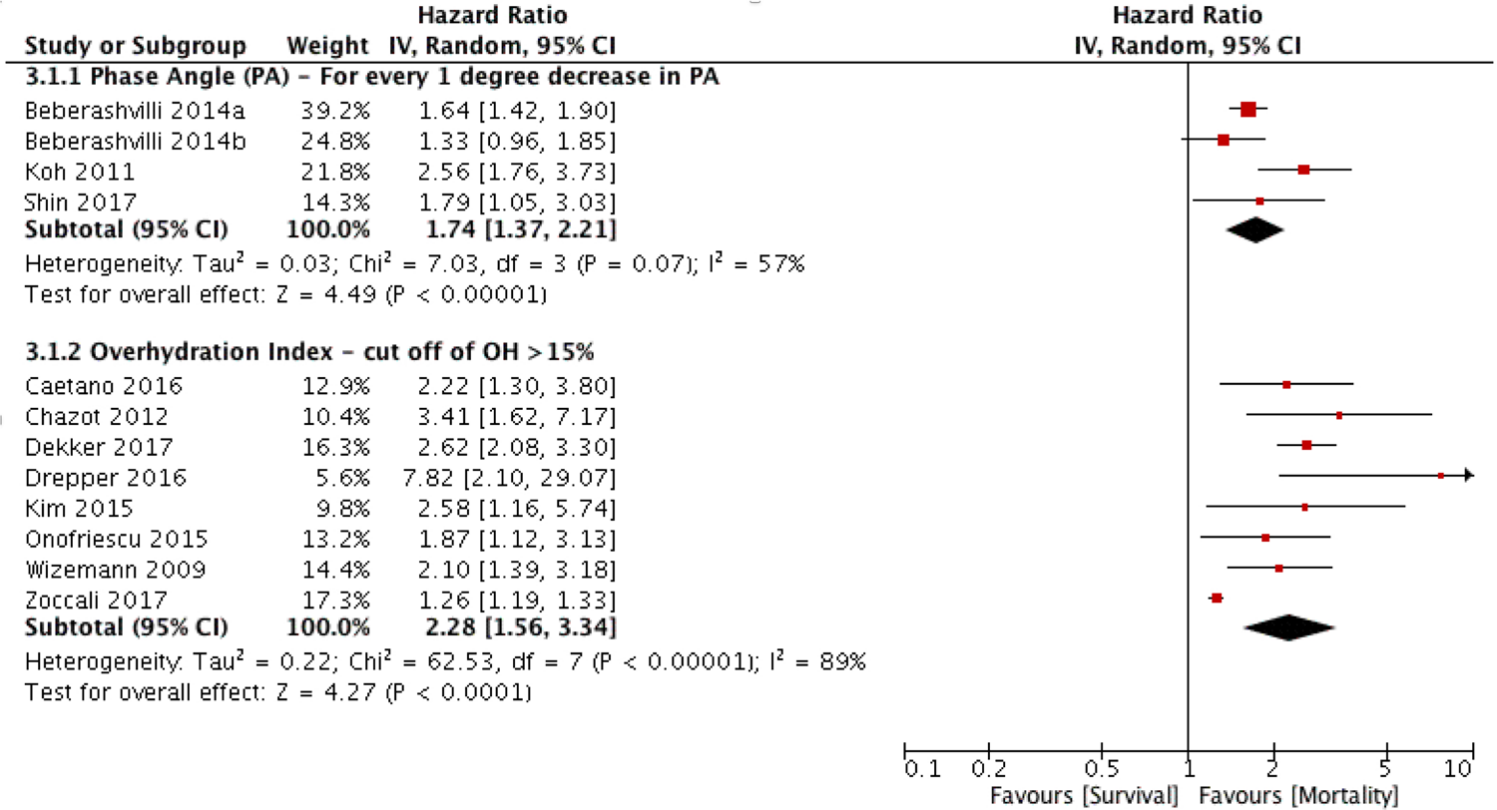
Summary of subgroup meta-analysis. The pooled summary of the effect of OH > 15% and a 1-degree decrease in PA on mortality in the dialysis population. 95% CI = 95% confidence interval, IV = inverse variance method.

Within 38 ESKF cohorts containing a MVSA, 32 reported adjusted hazard or odds ratio data for mortality for BI-OH measures along with 95% confidence intervals. Of these, 12 cohorts were eligible for meta-analysis (^1,2,15–17,23,35,37,38,44,54,61^, Table 2); 8 cohorts using an overhydration index cut off of OH/ECW > 15% and 4 cohorts using PA as a continuous variable. A 1-degree decrease in PA (HR 1.74, 95% CI 1.37–2.21, P < 0.001) and OH > 15% (HR 2.28, 95% CI 1.56–3,34, P < 0.001) were both predictive of mortality (Fig. 3), supporting the findings from the narrative synthesis of the evidence (Tables 1 and 2).

**Table 2:** Summary of cohorts reporting multivariate analyses with a stated hazard ratio, (HR) risk ratio (RR) or odds ratio (OR), 95% confidence intervals, (CI) lower limit (LL) and upper limit (UL).

Author(s)	Year	MVA Type	Mortality	Censored	BIA Marker	BIA Marker MVA	HR/OR for BIA	95% CI LL	95% CI UL	Reason for exclusion from MA
**End-stage Kidney Failure Cohorts**										
Demirci	2016	Cox analysis	93	Y^1,2,3^	BIVA	Impedance ratio	HR = 1.13	1.04	1.23	Only study using impedance ratio for BIVA analysis.
Pillon/Chertow	2004	Unclear MVA	361	Y^2,3,4,5^	BIVA	MVA using vector length (per 100ohm/m change)	RR = 0.75	0.57	0.88	Only 1 study using this BIA method
Chen	2007	Cox analysis	58	Y^1,2,3^	ECW Ratio	ECW/ICW - for every increase by 0.1 (time dependent)	RR = 1.37	1.1	1.7	Only study expressing continuous ECW/ICW variable in 0.1 increments.
Fan	2015	Cox analysis	37	N	ECW Ratio	ECW as an absolute value (in litres)	HR = 2.98	1.4	7.3	Only study expressing ECW as absolute volume.
Kim	2017	Cox analysis	24	Y^2^	ECW Ratio	ECW/ICW - for every increase by 0.01	HR = 1.12	1.01	1.25	Only study expressing continuous ECW/ICW variable in 0.01 increments.
Paniagua	2010	Cox analysis	182	N	ECW Ratio	ECW/TBW as continuous variable in CV mortality	OR = 1171.33	3.35	409899.37	Only study expressing ECW/TBW as continuous variable (expressed per unit ratio)
Rhee	2015	Cox analysis	15	N	ECW Ratio	ECW/TBW > Median	HR = 1.001	1.001	1.086	Only study expressing ECW/TBW > median
Guo/Guo	2015	Cox analysis	52	Y^1,2,3,4^	ECW Ratio	ECW/TBW > 0.4	HR = 13.12	1.35	128	Only study expressing ECW/TBW > 0.4 as cut off
Tian	2016	Cox analysis	44	N	ECW Ratio	ECW Ratio > 1 standard deviation from expected	HR = 2.20	0.79	6.08	Only study expressing ECW ratio > 1 standard deviation from expected
O’Lone	2014	Cox analysis	95	N	ECW Ratio+OH Index	Two markers: OH/ECW and ECW/TBW (highest 30% each)	HR 2.09 (1.36, 3.20)/HR 2.05 (1.31, 3.22)			Only study expressing both indices with 30% highest decile cut off
Caetano*	2016	Cox analysis	66	Y^1,2^	OH Index	OH/ECW > 15%	HR = 2.22	1.29	3.79	More than 1 study measuring OHI > 15%
Chazot*	2012	Cox analysis	Unclear	Y^2,4^	OH Index	OHI > 15% (dHS/ECW)	HR = 3.41	1.62	7.17	More than 1 study measuring OHI > 15%
Dekker*	2017	Cox analysis	Unclear	Y^1,2.3,4^	OH Index	OHI/ECW > 15% (overhydration 2.5–5L)	HR = 2.62	2.1	3.3	More than 1 study measuring OHI > 15%
Hoppe	2015	MLR	Unclear	N	OH Index	Continuous variable	OR = 1.12	0.92	1.37	Only study expressing OHI as continuous variable in MLR
Huan-Sheng	2016	Cox analysis	13	N	OH Index	Absolute OH; BIA-defined protocol linked with episodes of absolute fluid overload to determine management				RCT - testing an intervention vs. control, non comparable design.
Jotterand-Drepper*	2016	Cox analysis	19	Y^1,2,3^	OH Index	OHI/ECW > 15%	HR = 7.82	1.1	29.07	More than 1 study measuring OHI > 15%
Kim*	2015	Cox analysis	50	Y^2^	OH Index	OH/ECW > 15%	HR = 2.58	1.16	5.75	More than 1 study measuring OHI > 15%
Mathew	2015	MLR	41	Y^2,3^	OH Index	Absolute OH > Median (3.1L)	OR = 2.96	1.04	8.46	Only study expressing OHI > median
Onofriescu	2014	Cox analysis	9	Y^2,3^	OH Index	OH/ECW > 15% used to define BIA-defined overhydration in RCT of BIA-driven vs standard care.				RCT - testing an intervention vs. control, non comparable design.
Onofriescu*	2015	Cox analysis	66	Y^1,2,3,4^	OH Index	RFO (OH/ECW) > 15% and > 17.4%	15%: HR 1.87	1.12	3.13	More than 1 study measuring OHI > 15%
Siriopol/Siriopol	2015	Cox analysis	31	Y^1,2,4^	OH Index	OH/ECW > 6.68%	HR = 2.93	1.3	6.58	Only study expressing OHI > 6.68%
Siriopol	2017	Cox analysis	89	Y^1,2,4^	OH Index	OH/ECW > 6.9%	HR = 1.34	0.67	2.68	Only study expressing OHI > 6.9%
Tangvorap-honkchai	2016	Cox analysis	110	N	OH Index	OH as a continuous variable	HR = 1.15	1.03	1.28	Only study expressing OHI as a continuous variable in Cox regression.
Wizemann*	2009	Cox analysis	86	Y^2,4^	OH Index	OH/ECW > 15%	HR = 2.10	1.39	3.18	More than 1 study measuring OHI > 15%
Zoccali*	2017	Cox analysis	5866	N	OH Index	OH/ECW > 15% in males and > 13% in females	HR = 1.26	1.19	1.33	More than 1 study measuring OHI > 15%
Bebera-shvili**	2014	Cox analysis	38	N	PA	1 degree increase PA (time varying risk)	HR = 0.61	0.53	0.71	More than 1 study expressing PA as continuous variable
Bebera-shvili**	2014	Cox analysis	64	Y^2,3^	PA	PA - continuous variable in MVA	HR = 0.72	0.54	0.96	More than 1 study expressing PA as continuous variable
Fiedler	2009	Cox analysis	36	Y^2^	PA	PA < 4	HR = 2.34	1.O6	5.14	Only study expressing PA < 4
Koh**	2011	Cox analysis	35	N	PA	PA - continuous variable in MVA	HR = 0.39	0.27	0.57	More than 1 study expressing PA as continuous variable
Segall/Segall	2014	Cox analysis	11	N	PA	PA < 5.58	HR = 2.15	1.16	3.99	Only study expressing PA < 5.58
Shin**	2017	Cox analysis	15	N	PA	PA - continuous variable in MVA	HR = 0.56	0.33	0.97	More than 1 study expressing PA as continuous variable
de Araujo	2013	Cox analysis	13	Y^1,2,4,6^	PA+ECW Ratio	Stratified for diabetic status - PA predictive in nonDM/not predictive in DM				Stratified for diabetic status with two separate analyses
**Heart Failure Cohorts**										
Trejo-Velasco	2016	Cox analysis	19	N	BIVA	BIVA Hyperhydration (defined as > 74.3%)	HR = 2.60	1.10	6.40	Only study expressing BIVA
Sakaguchi	2015	Cox analysis	37	N	ECW Ratio	ECW Ratio (measured/predicted)	HR = 1.48	1.20	1.83	Only study expressing ECW ratio (measured/predicted)
Alves	2016	Cox analysis	34	N	PA	PA < 4.8	HR = 2.67	1.21	5.89	Only study expressing PA < 4.8
Colin-Ramirez	2012	Cox analysis	66	N	PA	PA < 4.2	HR = 3.08	1.06	8.99	Only study expressing PA < 4.2

Authors highlighted with * or ** had their studies included within the final subgroup meta-analysis. Censoring, where used within MVSA, are stated, with reasons including: Transfer to another RRT modality (1), transplantation (2), loss to follow up (3), transfer to another dialysis facility (4), withdrawal from RRT (5) or, in the case of one paper death due to non-cardiovascular cause (6).

### Methodological quality of studies

**Figure 4:**
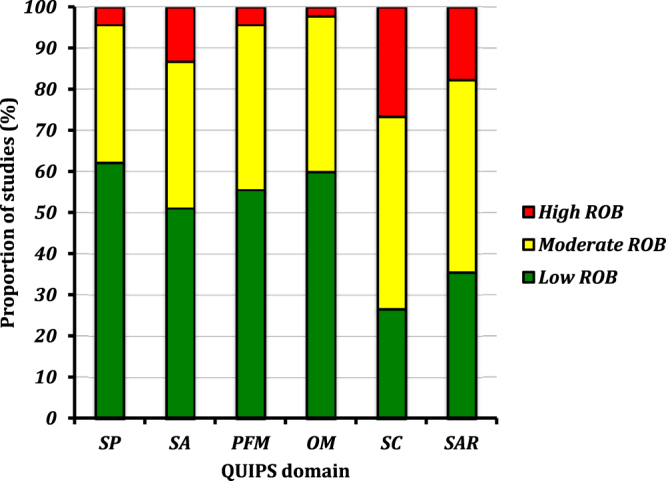
Summary of the QUIPS analysis from all cohorts included within the systematic review. SP = study participation, SA = study attrition, PFM = prognostic factor measurement, OM = outcome measure, SC = study confounding and STR = statistical analysis reporting.

Methodological quality varied widely between studies. Where more than one study was present within a cohort, the study where data was extracted for the review was appraised for methodological quality. Within all studies (Fig. 4), the majority of studies when assessed for ROB in study participation (62%^2,12,13,15,16,18,19,21,23–25,27,29,31,32,35,36,38,42,44,46,51–59,61^), study attrition (51%^1,2,15–17,19–24,30–32,35,36,39,40,42,44,45,47,51,57,58^), prognostic factor measurement (56%^1,2,13,15–17,20,21,24,26,28,31,32,37–41,47,49–51,58–61^) and outcome measurement (60%^1,12,13,15,16,19,21,22,24,27,30–32,35–42,44,45,49,51,55–57,60^) were deemed low ROB. In contrast, when assessing ROB in study confounding and statistical analyses, only 27%^2,12,17,19,22,25,38,44,46,52,53,58,61^ and 36%^15,22,26,27,30–32,40,41,44,45,49,51–53,57,58,61^ of studies had low ROB respectively. At the individual study level (Table 1) no study was rated as low risk or high risk of bias (ROB) in all six domains.

## Discussion

This systematic review provides strong narrative evidence, supported by quantitative evidence from a subgroup meta-analysis, that bio-impedance defined overhydration (BI-OH) is an independent predictor of mortality in ESKF patients. It is the first systematic review exploring this question and the first to demonstrate that different BI-OH metrics, such as phase angle or overhydration index (OHI), act as similar predictors of outcome, with overhydration defined by PA or OHI in the subgroup meta-analysis being associated with approximately double the risk of mortality compared with normohydrated patients. Furthermore, this is the first review to demonstrate that whole body BI-OH is an independent predictor of mortality in HF, suggesting a role for overhydration as a useful prognostic marker across different chronic disease groups.

BI-OH remained independently predictive of mortality or hospitalisation in all ESKF cohorts following adjustment for body mass index (BMI;^1,17,23,24,40,43,52,53,61^), subjective global assessment (SGA^19,30,39,52,53^), normalised protein nitrogen appearance (nPNA^39,52,53^) and malnutrition inflammation score (MIS^16,30^). This suggests that the additional predictive value of BI-OH is not confined to its ability to identify lean body mass cachexia but that it is also identifying absolute or relative expansion of the extracellular fluid volume as an independent risk. The association of ECFv expansion with malnutrition is not new, having been observed previously, using gold standard techniques of volume measurement in populations with cachexia due to poverty-related starvation^62^. However there are a number of additional explanations for this in the ESKD population, some of which were adjusted for in studies included in this review. Chronic inflammation (c-reactive protein or interleukin-6) was adjusted for in multiple cohorts (Table 2) and was itself an independent predictor of mortality in 43% of cohorts, without nullifying BI-OH as a predictor of mortality. This association^63,64^ is likely explained by the observation that inflammation drives lean body mass cachexia, with such changes being potentially driven by translocation of bacterial endotoxins across an oedematous bowel wall in overhydrated ESKF patients^65,66^. Chronic inflammation also contributes towards hypoalbuminaemia, which in our cohorts was demonstrated to a predictor of mortality in ESKF in half of all cohorts adjusting for it in MVSA; a finding consistent with previous studies that suggest hypoalbuminaemia may contribute towards intradialytic hypotension in haemodialysis and extravascular tissue oedema in peritoneal dialysis^64,67,68^. And yet, as demonstrated with chronic inflammation, hypoalbuminaemia did not nullify the ability of BI-OH to predict mortality in most cohorts, again suggesting the influence of overhydration on mortality in ESKF is synergistic. Cachexia, inflammation and hypoalbuminaemia is a common triad in many chronic diseases^69^, including in chronic kidney disease^69,70^, supporting the argument that overhydration in chronic diseases, as opposed to a catabolic metabolism, is contributing to poor outcomes.

Echocardiographic abnormalities are common in ESKF; one previous study estimating the prevalence of left ventricular hypertrophy and systolic dysfunction in dialysis patients to be 74% and 15% respectively^71^. Our review demonstrates BI-OH remains an independent predictor of mortality even in the presence of abnormal left ventricular ejection fraction (LVEF^44^) and mass index (LVMI^56^). Although cautious interpretation is warranted given the small number of cohorts including echocardiogram data, our findings add weight to the developing narrative that cardiac structural disease, particularly left ventricular systolic dysfunction (LVSD), may not be the *sine qua non* underlying excess mortality in overhydrated ESKF patients. The link between BI-OH and adverse cardiovascular events has been previously noted; BI-OH previously being correlated with endothelial dysfunction^72^, arterial stiffness^43^ and the development of left ventricular hypertrophy (LVH^73,74^). In studies exploring sudden cardiac death in ESKF, LVH was predictive of mortality even when adjusting for blood pressure, whereas LVSD played no such role in predicting mortality. What cannot be answered by current evidence is whether LVH precipitates sudden cardiac death or whether LVH merely acts as a surrogate for overhydration, since the Onofriescu *et al*. study demonstrated improvements in LVH correlated with improvements in BI-OH measurements^43^. Furthermore, a recent systematic review and meta-analysis by Badve *et al*. suggests that in CKD, interventions to reduce LVH through altering volume status are not consistently effective, and even where they do reduce LVH (for example through improving haemoglobin or renin-angiotensin blockade), no survival benefit has been seen^75^. The role of BNP as a predictor of overhydration and mortality was explored in four cohorts and data from one suggested a role for both BNP and BI-OH as independent predictors of mortality, albeit echocardiography was not included in this analysis. One hypothesis is that mortality in overhydrated ESKF patients may be driven by the dialysis prescription^76^, with greater ultrafiltration rates during dialysis having been previously demonstrated to induce HD-induced cardiac injury in the form of regional wall motion abnormalities subclinical myocardial ischaemia^77,78^. However, given that two cohorts demonstrated a role for BI-OH and not BNP as independent predictors of mortality, there is still much to be learned about the interaction of cardiac biomarkers and overhydration in predicting outcomes in dialysis patients.

Two recently published large international studies are included in our review^23,61^. Dekker *et al*. demonstrated in a European multinational cohort using data from 5450 selected HD patients that baseline pre-dialysis BI-OH (where the definition of severe fluid overload was >2.5 L absolute overhydration) predicted increased mortality when adjusted for multiple demographic and co-morbidity covariates. Furthermore this study demonstrated an additive risk of mortality in overhydrated patients with chronic inflammation^23^. The second study, by Zoccali *et al*., demonstrated in an multinational cohort using data from 39,566 ESKF patients that when adjusted for multiple demographic and co-morbidity covariates that overhydration at baseline, defined as an OHI > 15% for men and >13% for women, is an independent predictor for mortality^61^. They also explored the well established J-shaped relationship between pre-dialysis blood pressure and mortality, finding that higher mortality in overhydrated compared with normohydrated patients is observed across all blood pressure strata, and demonstrated that overhydration remained an independent predictor of mortality with cumulative BI-OH measurements over a one year period^61^. The inclusion of these studies adds significant value to our narrative and pooled summaries, establishing that in approximately 50,000 dialysis patients baseline OHI > 15% is predictive of mortality despite adjustment for multiple comorbidities and inflammation.

Our review has several strengths, including the use of systematic methods to identify studies, independent reviewers throughout the study selection, review and quality appraisal process and the inclusion of heart failure studies as a comparator group, to explore the role of BI-OH in different chronic disease states. It is the first attempt to our knowledge, to summarise and compare the utility of different BI-OH measures in predicting mortality. The review does however have several limitations. Methodological heterogeneity within the studies was considerable, with common sources of bias including unclear study design^14,20,47,50^, inadequate reporting of cohort demographics^1,2,20,26,28,31,32,40,41,43–45,47,50^, inadequate description or insufficient numbers of endpoints^2,17,18,20,22,25,28,29,31,32,37,46,47,49–51,55,56^, lack of clarity regarding the protocol for the measurement of BI-OH^16,17,19,31–33,45,46,55,56^, exclusion of clinically relevant covariates from MVA^26,30–33,37,40^ and a lack of clarity regarding the statistical methods used during survival analysis^2,14,16,18–21,26,29,39,46,47^. In some studies there was a failure to adjust for HIV status in cohorts where prevalence of HIV is high or where the large proportions of the population is African-Americans;^20,22,29,38,47,49^ importantly BI-defined cachexia is associated with HIV infection and therefore potentially confounds the association of BI-OH and mortality. Finally considerable heterogeneity within BI-OH method reporting, and particularly the use of different BI devices which use different “normal populations” to define their BI-OH cut offs, limited the scope for performing a comprehensive pooled survival analysis. This particularly explains why all cohorts expressing BI-OH using the ECWR method could not be pooled, as they all depend on the algorithms used for total body water estimation, which differs between devices^3^ and is potentially confounded by ethnicity. Given the anticipated heterogeneity within our pooled analysis we followed the recommendation of Higgins *et al*. when planning our meta-analysis^79^, including the use of a random effects method, assessing for a consistent pattern in the directionality of the results in included studies and the use of studies which adjust for the effects of covariates on the outcome variable.

This review clearly establishes BI-OH as a predictor of survival in ESKF patients, independent of the effect of malnutrition, inflammation, multimorbidity and within a few cohorts, cardiac structural disease. Similar conclusions are noted in HF patients, suggesting a role for overhydration in predicting poor outcomes in other chronic diseases - a hypothesis which should be tested in other disease groups. The evidence presented necessitates further investigation into the pathogenic role of overhydration, for example through real-time cardiac imaging and ultrafiltration rate during dialysis or the prognostic value of BI-OH in preventing volume related deaths contributing to the increased mortality observed during the 3-day break. Likewise, it does not establish the value of BI-OH as a tool for goal directed fluid management. Although recent trials suggest that use of BI can improve fluid status and blood pressure, as summarised by Covic *et al*. in a systematic review and in the recent UK NICE guidelines^80–82^, with further studies are on-going^83^, there is no clear benefit on all-cause mortality.

## Electronic supplementary material

Search Strategy
