# Supplementary material for: Bioimpedance-defined overhydration predicts survival in end stage kidney failure (ESKF): systematic review and subgroup meta-analysis
Source: Sci Rep. 2018 Mar 13;8:4441. doi: 10.1038/s41598-018-21226-y (PMC5849723; doi:10.1038/s41598-018-21226-y)
Supplement: Supplementary file 1 — Search Strategy [file 41598_2018_21226_MOESM1_ESM.pdf]

Bioimpedance-defined overhydration predicts survival in end stage kidney failure (ESKF): systematic review and subgroup meta-analysis

Matthew Tabinor, Emma Elphick, Michael Dudson, Chun Shing Kwok,
Mark Lambie, Simon J Davies

Institute for Applied Clinical Sciences, Keele University, UK

Supplementary Material

(1) Supplementary Table 1

(2) Appendix Summary of the strategy used whilst searching CENTRAL.

Supplementary Table 1: Summary of co-variates included in multivariable survival analyses (MVSA) within

(a) End-Stage Kidney Failure and (b) Heart Failure Cohorts

(a) End Stage Kidney Failure Cohorts

					Co-morbidity matrix for covariates included in MVSA																														
Author(s)	*Year*	*Prim. Outcome*	*N(Mort)*	*MVA Type*	Censored	Age	Ethn	Gen	BMI	Modality	Vintage	Hospital stay	DM	P/CVD	Comorb	Chol	CRP	IL-6	HIV	CKD	NHYA	HF	BP	LVEF	Alt/Echo	BNP	Trop	HbA	KtV/Cr	Alb	Phos	D/PCr	NonBIA	RRF	Prev/Adm	*BI-OH Method*
Abad	2011	Mortality	100	Cox analysis	N	**X**		X			X																									**PA***
Avram	2006	Mortality	89	Cox analysis	N	**X**	X	X					X																							**PA***
Beberashvili	2014	Mortality	38	Cox analysis	N	**X**		X		X	X		X	X																						**PA****
Beberashvili	2014	Mortality	64	Cox analysis	Y	**X**		**X**		X	X		X		X			X											X				X	X		**PA*****
Caetano	2016	Mortality	66	Cox analysis	Y	**X**		X		X	X		X																							**OHI****
Chazot	2012	Mortality	Unclear	Cox analysis	Y			X	X	X	X												X													**OHI****
Chen	2007	Mortality	58	Cox analysis	Y	**X**		**X**							X														X	X	X		X			**ECWR****
de Araujo	2013	CV Events	13	Cox analysis	Y	**X**				X							X						X													**PA**
Dekker	2017	Mortality	Unclear	Cox analysis	Y	**X**		X	X		X		X	X								X											X			**OHI****
Demirci	2016	Mortality	93	Cox analysis	Y			**X**	X		X		X										X					X	X							**BIVA****
Di Iorio	2004	Mortality	75	Cox analysis	N																															**PA***
Fan	2015	Mortality	37	Cox analysis	N	**X**							X																	X						**ECWR****
Fein	2002	Mortality	4	Univariate analysis																																**PA******
Fein	2008	Mortality	21	Cox analysis	Y	**X**	X	**X**					X																							**ECWR***
Fiedler	2009	Mortality	36	Cox analysis	Y	X		X			X		X																							**PA****
Guo/Guo	2015	Mortality	52	Cox analysis	Y	X		X					X		X		X						X						X	X		X		X		**ECWR****
Hoppe	2015	Mortality	42	MLR	N								X			X									X		X									**OHI**
Huan-Sheng	2016	Hospitalisation	13	Cox analysis	N	X		X																												**OHI**
Jotterand-Drepper	2016	Mortality	19	Cox analysis	Y												X					X				X	X			X						**OHI****
Kim	2015	Mortality	50	Cox analysis	Y	X		X					X	X														X	X	X	X					**OHI****
Kim	2017	Mortality	24	Cox analysis	Y	X							X										X													**ECWR****
Koh	2011	Mortality	35	Cox analysis	N	X		X			X		X										X						X	X	X					**PA****

Maggiore	1996	Mortality	23	Cox analysis	N																															PA*
Mathew	2015	Mortality	41	MLR	Y				X																											OHI**
O'Lone	2014	Mortality	95	Cox analysis	N										X														X							OHI+ ECWR**
Oei	2016	Mortality	48	Univariate analysis																																OHI****
Onofriescu	2014	Mortality	9	Cox analysis	Y	X		X	X		X		X	X																						OHI**
Onofriescu	2015	Mortality	66	Cox analysis	Y	X		X			X		X	X									X	X	X											OHI**
Paniagua	2010	Mortality	182	Cox analysis	N	X		X		X	X		X										X				X									ECWR**
Paudel	2015	Mortality	72	Univariate analysis																																OHI****
Pillon/ Chertow	2004	Mortality	361	Unclear	Y	X	X	X			X		X			¶												X	¶	X						BIVA**
Ponce	2014	Mortality	20	Univariate analysis																																OHI
Pupim	2004	CV Mortality	23	Cox analysis	Y	X	X	X									X												X							PA*
Rhee	2015	Residual RF	15	Cox analysis	N	X		X					X																							ECWR**
Segall/ Segall	2014	Mortality	43	Cox analysis	N																	X														PA**
Shin	2017	Mortality	15	Cox analysis	N	X		X							X																					PA**
Siriopol/ Siriopol	2015	Mortality	31	Cox analysis	Y	X					X						X				X				X					X						OHI**
Siriopol	2017	Mortality	89	Cox analysis	Y			X			X															X										OHI
Tangvoraphonkchai	2016	Mortality	110	Cox analysis	N	X																				¶										OHI**
Tian	2016	Mortality	44	Cox analysis	N	X											X						X							X			X			ECWR
Wizemann	2009	Mortality	86	Cox analysis	Y			X			X																		X	X	X					OHI**
Zoccali	2017	Mortality	5866	Cox analysis	N				X				X															X	X		X		X			OHI**

(b) Heart Failure Cohorts																																				
					Co-morbidity matrix for covariates included in the MVSA																															
Author(s)	***Year***	***Prim. Outcome***	***N (Mort)***	***MVA Type***	**Censored**	**Age**	**Ethn**	**Gen**	**BMI**	**Modality**	**Vintage**	**Hospital stay**	**DM**	**P/CVD**	**Comorb**	**Chol**	**CRP**	**IL-6**	**HIV**	**CKD**	**NHYA**	**HF**	**BP**	**LVEF**	**Alt/Echo**	**BNP**	**Trop**	**HbA**	**KtV/Cr**	**Alb**	**Phos**	**D/PCr**	**NonBIA**	**RRF**	**Prev/Adm**	***BI-OH method***
Alves	2016	Mortality	34	Cox analysis	N	**X**																														**PA****
Castillo-Martinez	2007	NYHA Class	N/A	Univariate analysis																																**PA+BIVA******
Colin-Ramirez	2012	Mortality	66	Cox analysis	N								**X**																							**PA****
Doesch	2010	Comparison with CMR.	8	Univariate analysis																																**PA**
Sakaguchi	2015	EF Survival	37	Cox analysis	N	**X**														**X**				**X**		**X**		**X**							**X**	**ECWR****
Trejo-Velasco	2016	Adverse Events	37	Cox analysis	N	**X**														**X**																**BIVA****

Supplementary Table 1. Abbreviations and key: N (Mort) = number of endpoints (Defined in table 1). Covariates included in the MVSA are identified in the matrix: covariates coded “ ” are included in the MVSA and independently predict the primary outcome, “X” indicates they do not predict outcome, “¶” indicates in multi-study cohorts that the covariate was only included in 1 study and was not predictive of the primary outcome and blank squares denote the covariate was not included. BI-OH = type of measure used as described with abbreviations in text. MLR = Multiple Linear Regression, Ethn = ethnicity, Gen = gender, BMI = Body Mass Index, DM = diabetes mellitus, CVD = cardiovascular disease, Comorb = comorbidity Index (e.g. Charlson index), Chol = cholesterol, NHYA = NHYA classification of dyspnoea, HF = heart failure, LVEF = left ventricular ejection fraction, Alt/Echo = non LVEF echocardiographic measurements, BNP = brain natriuretic peptide, Trop = Troponin, HbA = Haemoglobin / Ferritin, KtV = KtV or creatinine, Alb = albumin, Phos = phosphate, PSTR = peritoneal solute transport rate, NonBI Nut = Other nutritional markers (i.e. SGA), RRF = residual renal function, Prev Adm = previous admission to hospital.

Appendix: summary of the strategy used whilst searching CENTRAL.

Each individual term is numbered, with the number of results for each search term being given at the end in parentheses.

Initial Search Process	
MeSH	Individual Search Terms
(1) “Renal Dialysis” (4417)	(5) “dialysis” or “hemodialysis” or “haemodialysis” or "peritoneal dialysis" or "renal replacement therapy" (11964)
(2) “Chronic Kidney Disease” (3481)	(6) "end stage renal failure" or “esrf” or "end stage renal disease" or “esrd” or "chronic kidney disease" or “ckd” or "renal failure" or "kidney failure" or "renal insufficiency" or "kidney insufficiency" or "renal injury" or "kidney injury" (13450)
(3) “Electrical Impedance” (360)	(7) “bioimpedance” or "bio-impedance" or "bioimpedance analysis" or "bio-impedance analysis" or “bia” or "bio-impedance vector analysis" or "bioimpedance vector analysis" or “biva” or "phase angle" or "extracellular water" or "electrical impedance" (824)
(4) “Heart Failure” (5903)	(8) "heart failure" or "congestive cardiac failure" or “ccf” or "left ventricular systolic dysfunction" or “lvsd” or "left ventricular diastolic dysfunction" or “lvdd” or "right ventricular systolic dysfunction" or “rvsd” or "right ventricular diastolic dysfunction" or “rvdd” or "right sided heart failure" or "left sided heart failure" or "biventricular heart failure" or "right-sided heart failure" or "left-sided heart failure" or "bi-ventricular heart failure" (16047)
**Combining Search Terms**	

(9) MeSH terms: (1) or (2) or (4) and (3) – 31 studies
(10) Individual terms: (5) or (6) or (8) and (7) - 121 studies
(11) Final search output: (9) or (10) – 128 studies
(12) When cut off from 1990 and selected for manuscripts available in English language – **120 studies**
